# Perception of Malaysian primary care practitioners towards patients with substance use disorders in Selangor and its associated factors: A cross-sectional study

**DOI:** 10.51866/oa.845

**Published:** 2026-02-26

**Authors:** Shamsul Bahrin Nasehah Sakeenah, Aneesa Abdul Rashid

**Affiliations:** 1 Klinik Kesihatan Buloh Kasap, Jalan Tasik Alai, Segamat, Johor, Malaysia.; 2 Department of Family Medicine, Universiti Putra Malaysia, Serdang, Selangor, Malaysia.

**Keywords:** Substance use disorder, Attitudes, Healthcare providers, Drug abuse, Health personnel attitudes

## Abstract

**Introduction:**

Substance use disorders (SUDs) represent a significant public health concern, with an estimated 36.3 million people affected worldwide and approximately 100,000 active drug users in Malaysia. As individuals with SUDs frequently disengage from treatment, the cultivation of non-judgemental attitudes among healthcare professionals is critical. This study aimed to assess the perceptions of primary care practitioners (PCPs) in Selangor towards patients with SUDs and identify the associated factors.

**Methods:**

A cross-sectional study was conducted from June 2021 to November 2023 among 235 PCPs in Selangor, utilising convenience sampling. Data were collected via an online, self-administered questionnaire. Perceptions were measured using the Drug and Drug Problems Perception Questionnaire (DDPPQ) with lower scores indicated more positive perceptions. Multiple linear regression was employed to identify the associated factors.

**Results:**

The participants were predominantly women (76.8%) and medical officers (46.8%), with a median experience of 8 years. While the majority (73.4%) reported clinical encounters with patients with SUDs, only 24% had received specific SUD training and 23.2% had experience in methadone maintenance therapy (MMT). The mean total DDPPQ score was 45.38±6.91 reflecting generally negative perceptions. The regression model (R^2^=0.268) showed that favourable perceptions were significantly associated with prior MMT experience (B=-4.91, P<0.001) and higher frequency of clinical interaction (e.g. never vs always interaction: B=12.53, P<0.001).

**Conclusion:**

The PCPs in this study exhibited a predominantly negative perception towards patients with SUDs. More negative perceptions were significantly associated with a lack of experience in MMT and less frequent clinical contact with this patient population.

## Introduction

Substance use disorder (SUD) is a multifaceted issue characterised by an excessive and unregulated consumption of a substance, despite negative consequences.^1^ Individuals with SUDs develop a strong preoccupation with consuming a specific substance such as tobacco, alcohol or illegal drugs, to the extent that it impairs their ability to function effectively in their daily lives. Despite being aware that their substance use causes or will cause problems, people with SUDs continue to consume the substance.

SUD remains a problem in most countries. Globally, approximately 36.3 million people experienced SUD in 2019, showing an increment of six billion people as previously reported in 2016.^2^ In addition, there were 494,000 deaths attributed to drug use reported in 2019.^2^ Furthermore, 275 million people worldwide aged 15–64 years are estimated to have used drugs at least once in the previous year, equivalent to 1 in every 18 people within that age group.^2^ Conversely, Asia has the largest number of people who inject drugs globally, amounting to 5.2 million.^3^

In Malaysia, approximately 300,000 people have used drugs once in their lifetime, as reported in the National Health and Morbidity Survey in 2019, and 100,000 people actively use them.^4^ The majority of substance abusers and addicts in Malaysia are Malay (78.8%), with 95.7% of them being men. The highest number of people with SUDs was noted among those aged 19–39 years, accounting for 65.9% of the overall prevalence. Moreover, Selangor had the third highest number of people with SUDs in 2021, as reported by the National Anti-Drug Agency.^6^ The highest prevalence of SUD in Malaysia was noted in Kedah (11%), Johor (10.8%) and Selangor (9.9%), with 13,261, 13,502 and 12,182 people with SUDs, respectively. Thus, with the rising prevalence of SUDs globally, primary care providers (PCPs), as the initial points of contact within the healthcare system, play a pivotal role in the early identification of affected individuals and facilitation of timely referral to appropriate treatment services.

Van Boekel et al.^7^ conducted a systematic review of 28 Western studies on stigma among health professionals towards patients with SUDs. Their study revealed that healthcare workers generally held a negative perception towards patients with SUDs, and this resulted in suboptimal healthcare delivery towards them. Factors such as violence, manipulation and poor motivation were identified as obstacles in delivering healthcare to these patients.^7^ Furthermore, healthcare workers lacked sufficient education, training and support structures to effectively work with this specific patient group.^7^ These negative perceptions of health professionals had a detrimental impact on patients’ sense of empowerment and subsequent treatment outcomes.^7^ Health professionals displayed less involvement and a more task-oriented approach, leading to reduced personal engagement and diminished empathy. In Asia, studies have shown that healthcare professionals who are directly involved in the management of patients with SUDs on a daily basis also tend to exhibit negative perceptions towards them. For example, both Norsiah et al.^8^ and Tang et al.^9^ explored the perceptions of healthcare professionals who were working directly with patients with SUDs. Norsiah et al.^8^ found that stigma towards methadone therapy was evident among staff handling methadone maintenance therapy (MMT) clinics and suggested for more awareness programmes and training on local methadone guidelines to be conducted for them. Tang et al.^9^ found that participants generally had negative perceptions and exhibited a punishment-oriented philosophy, with only approximately half feeling that substance abuse should be treated as a brain disorder. The authors also found that personnel generally lacked sufficient qualifications, training and experience in the field of drug abuse treatment. In community settings, both Li et al.^10^ and Lin et al.^11^ showed that community health workers (CHWs) including doctors, assistant doctors, nurses, midwives and pharmacists exhibited higher levels of stigma towards patients with SUDs than towards people living with HIV. CHWs reported perceiving difficulties in establishing contact with individuals who have SUDs, expressed challenges in ensuring adherence to treatment among them and indicated feelings of discouragement when patients were found to be dishonest.

Although previous studies have examined healthcare professionals’ perceptions towards individuals with SUDs, there is a lack of research specifically addressing these perceptions within the Malaysian primary care setting. Existing studies in Malaysia have largely focused on prevalence, treatment outcomes or public attitudes, with limited attention given to the attitudes and perceptions of frontline healthcare providers, particularly PCPs. Furthermore, the factors influencing these perceptions such as training exposure, professional background and clinical experience with patients with SUDs remain underexplored in the local context. This is concerning given the rising prevalence of substance use and misuse in Malaysia, which underscores the need for responsive and empathetic care at the primary care level. Therefore, this study aimed to assess the perceptions of PCPs, including doctors, assistant medical officers and nurses, towards patients with SUDs in Selangor and identify the factors associated with these perceptions.

## Methods

### Study design and setting

This cross-sectional study was conducted from June 2021 to November 2023. It involved PCPs, including doctors, nurses and assistant medical officers, working in primary care clinics in Selangor, Malaysia. Licensed PCPs who had worked in a primary care setting for at least 6 months were included in the study. Data were collected using an online questionnaire administered via Jotform.

### Sample size and sampling

The sample size was calculated using a web calculator from Arifin^12^ and the two-sample proportion formula,^13^ with a medium effect size of 0.5 (two-sided), significance level of 0.05 and power of 80%. The figures were chosen based on a previous study on mental health professionals’ attitude and perception of their role in tackling substance abuse and related disorders in Nigeria.^14^ Given a 20% non-response rate, the sample size calculated was 234. The study employed a convenience sampling approach, which is cost-effective, efficient and straightforward to execute; however, the sample may lack clear generalisability.^15^ Selecting a probability-based sampling method was not feasible due to the following reasons: The timeframe for data collection was relatively brief, and the intended population was difficult to reach.

### Study tools

The questionnaire had three parts. Part 1 contained questions regarding participants’ sociodemographic profile including age, sex, educational level, smoking status, alcohol use and family members with drug use problems or undergoing treatment for such problems. Part 2 comprised questions regarding professional practice. It was divided into the following three categories: drug and alcohol use training, professional experience and interaction with the patient group. Part 3 included the Drug and Drug Problems Perception Questionnaire (DDPPQ) by Watson et al.^16^ in both English and Malay languages.

The DDPPQ is a concise, valid and reliable tool for assessing therapeutic attitudes, with strong internal consistency (Cronbach’s a=0.87). Since its development, it has been widely used, including adaptations for adolescents and cross-cultural validations in various countries and languages. Notably, it has been translated and validated in Japan (J-DDPPQ), showing excellent internal consistency (a=0.92) and construct validity. Given its established psychometric robustness and successful application in the Asian context, the DDPPQ was deemed appropriate for use in this study in Malaysia, with cultural adaptation as necessary.^14,17-22^

The Malay version, termed DDPPQ-M, was developed through a rigorous back-to-back translation method to ensure conceptual and semantic equivalence with the original instrument. Translated items were subsequently reviewed by an expert panel comprising two family medicine specialists and two family medicine trainees. Items demonstrating the closest alignment with the original DDPPQ were selected for inclusion in the final version of the DDPPQ-M.

A pilot study involving 30 participants from Klinik Kesihatan Presint 9, Putrajaya, was conducted to evaluate item clarity and overall comprehensibility. Participant feedback was systematically analysed to enhance content validity. Reliability testing of the DDPPQ-M yielded a high internal consistency, with an overall Cronbach’s a of 0.925. Subscale reliability coefficients ranged from 0.833 to 0.952, indicating robust internal reliability across all dimensions of the instrument.

The DDPPQ-M contained 20 items and measured five subscales: role adequacy, role legitimacy, role support, role-related self-esteem and job satisfaction. The scoring of the DDPPQ was adapted from the study by Akinola,^14^ whereby all items were attached to 4-point Likert scale responses and scored from 1 *(strongly agree)* to 4 *(strongly disagree)* to avoid neutral responses and central tendency bias without affecting the psychometric properties. Perception was measured by summing up all item scores in each subscale of the DDPPQ, with mean scores calculated. Lower overall scores denoted more positive perceptions, while higher scores denoted more negative views:

1. Role adequacy: seven items (possible score: 7-28)
2. Role legitimacy: two items (possible score: 2-8)
3. Role support: three items (possible score: 3-12)
4. Role-related self-esteem: four items (possible score: 4-16)
5. Job satisfaction: four items (possible score: 4-16)

The scores of the DDPPQ and its subscales were then interpolated and tabulated for comparative purposes,^23-25^ with the mean and standard deviation (SD) of the total DDPPQ score calculated out of 140.

### Data analysis

The data were analysed using the Statistical package for the Social Sciences software (version 29.0, IBM Corp., Armonk, New York, NY, USA). The normality of numerical variables was assessed using visual (histograms and Q–Q plots) and statistical (Kolmogorov–Smirnov and Shapiro–Wilk tests) methods. As different variables were tested for normality independently, both parametric and non-parametric tests were used as appropriate for each variable’s distribution. Normally distributed data were reported as means ± SDs and non-normally distributed data as medians and interquartile ranges (IQRs). Bivariable analysis included independent t-tests, the Mann–Whitney U test, the Kruskal–Wallis H test and Spearman’s rank-order correlation. Variables with a P-value of <0.25 in the bivariable analysis were entered into a multiple linear regression model to identify the significant predictors of PCPs’ perceptions. A P-value of <0.05 was considered statistically significant.

## Results

The median age of the participants was 37 years (IQR=7). Most participants were women (76.8%, n=179), held a degree (45.1%, n=105), did not smoke or drink any alcohol (96.6%, n=225) and had no family members with SUDs (87.6%, n=204). The majority were medical officers (46.8%, n=109), with a median working experience in the primary care setting of 8 years (IQR=7). Approximately 76% (n=177) of the participants did not receive any specific training regarding alcohol and drug use problems. Only 23.3% (n=54) had experience with MMT, 14.2% (n=33) with Alcoholics/Narcotics Anonymous and 7.7% (n=18) with detoxification programmes. The median duration of training received since the past 12 months was 6 hours (IQR=8). The majority of the participants had seen patients with alcohol and/or other drug use problems during their service in primary care (73%, n=171) (Table 1).

**Table 1:** Sociodemographic characteristics and DDPPQ scores (N=233).

Variable	n (%)	Median (IQR)
**Age, year**		37 (7)
**Sex**		
Male	54 (23.2)	
Female	179 (76.8)	
**Educational level**		
Certificate	24 (10.3)	
Diploma	75 (32.2)	
Degree	105 (45.1)	
Master’s degree	28 (12.0)	
PhD	1 (0.4)	
**Smoking**		
Yes	8 (3.4)	
No	225 (96.6)	
**Alcohol consumption**		
Yes	8 (3.4)	
No	225 (96.6)	
**Family members with SUDs**		
Yes	18 (7.7)	
Not sure	11 (4.7)	
No	204 (87.6)	
**Profession**		
Medical officer	109 (46.8)	
Family medicine specialist	20 (8.6)	
Staff nurse	63 (27.0)	
Assistant medical officer	21 (9.0)	
Jururawat masyarakat	20 (8.6)	
**Working experience in primary care**		8 (7)
**Received specific training regarding alcohol and other drug use problem**		
Yes	56 (24)	
No	177 (76)	
**Experience with MMT**		
Yes	54 (23.2)	
No	179 (76.8)	
**Experience with detoxification programmes**		
Yes	18 (7.7)	
No	215 (92.3)	
**Experience with Alcoholics or Narcotics Anonymous**		
Yes	33 (14.2)	
No	200 (85.8)	
**Interaction with patient groups**		
Yes	171 (73.4)	
No	62 (26.6)	
**Frequency of seeing patients with SUDs**		
Never	49 (21.0)	
Rarely	79 (33.9)	
Sometimes	84 (36.1)	
Often	14 (6.0)	
Always	7 (3.0)	
	**Mean score out of 80 (SD)**	Mean score out of 140 (SD)^*^
Total DDPPQ score (possible score: 7–80)	45.38 (6.91)	70.76 (13.82)

* Scores were interpolated and tabulated for comparative purposes. Lower scores denoted more positive perceptions.

DDPPQ: Drug and Drug Problems Perception Questionnaire IQR: Interquartile range MMT: Methadone maintenance therapy SUD: Substance Use Disorder SD: Standard deviation

### Perception towards patients with SUDs

We found that the mean total DDPPQ score of the participants was 45.38 (SD=6.91) out of 80, with a range of 21–67, showing a generally negative perception. The scores of the DDPPQ and its subscales were then interpolated and tabulated for comparative purposes.^23-25^ The mean total DDPPQ score out of 140 was 70.76 (SD=13.82) (Table 1).

### Factors associated with the perception towards patients with SUDs

Table 2 summarises the results of the bivariable analysis of the factors associated with the total DDPPQ score. The factors with a P-value of <0.25 were included in the multiple regression model.

**Table 2:** Factors associated with the total DDPPQ score (N=233).

Variable	n	Mean (SD)	Median (IQR)	P-value
**Sex**				0.013^d^^*^
Male	54	43.35 (7.74)^d^	44 (8)	
Female	179	45.99 (6.54)^d^	45(8)	
**Educational level**				<0.001^b^^**^
Certificate/diploma	99		46 (9)	
Degree	105		45 (8)	
Master’s degree/PhD	29		41 (8)	
**Profession**				<0.001^b^^**^
Medical officer	109		45 (9)	
Family medicine specialist	20		40.5 (9)	
Staff nurse	63		47 (9)	
Assistant medical officer	21		44 (6)	
Jururawat masyarakat	20		48.5 (11)	
**Family members with SUDs**				0.132^b^
Yes	18		41.5 (11)	
Not sure	11		45 (7)	
No	204		45 (7)	
**Received training on alcohol and drug use**				<0.001^d^^**^
Yes	56	40.61 (7.51)^d^	41 (9)	
No	177	46.89 (5.98)^d^	47 (8)	
**Duration of training since the past 12 months**			6 (8)	0.166^c^
**Experience with MMT**				<0.001^d^^**^
Yes	54	40.13 (6.99)^d^	41 (8)	
No	179	46.97 (6.07)^d^	47 (8)	
**Experience with detoxification programmes**				0.004^d^^**^
Yes	18	40.89 (7.05)^d^	42.5 (8)	
No	215	45.76 (6.78)^d^	45 (8)	
**Experience with Alcoholics or Narcotics Anonymous**				<0.001^d^^**^
Yes	33	40.55 (6.96)^d^	42 (7)	
No	200	46.18 (6.59)^d^	46 (7)	
**Seen patients with alcohol/drug use problem during service**				0.001^d^^**^
Yes	171	44.44 (6.67)^d^	44 (8)	
No	62	47.97 (6.95)^d^	47.5 (9)	
**Frequency of seeing patients with SUDs**				<0.001^b^^**^
Never	49		49 (10)	
Rarely	79		46 (7)	
Sometimes	84		44 (7)	
Often	14		42 (11)	
Always	7		32 (18)	

* P<0.05

** P<0.001

Bolded items: Items with P<0.25 that were chosen to be included in the multiple regression analysis

b Kruskal–Wallis H test

c Spearman’s rank-order correlation

d Independent t-test

DDPPQ: Drug and Drug Problems Perception Questionnaire IQR: Interquartile range MMT: Methadone maintenance therapy SUD: Substance Use Disorder SD: Standard deviation

### Predictors of the perception towards patients with SUDs

The final multiple linear regression model (Table 3) was statistically significant and accounted for 26.8% of the variance in the total DDPPQ score (R^2^=0.268). There was no evidence of multicollinearity (VIF<1.170). The two significant predictors were experience with MMT and frequency of seeing patients with SUDs. The participants with experience with MMT had significantly lower DDPPQ scores (indicating a more positive perception) than those with no experience (B=-4.91, P<0.001). The 95% confidence interval (CI) (-6.90, -2.91) did not exceed 0, providing strong evidence of a reliable association. As for the frequency of seeing patients with SUDs, using the *always* group as the reference, a lower frequency of interaction was associated with higher DDPPQ scores (more negative perceptions). The effect was strongest for the *never* group (B=12.53, P<0.001; 95% CI [7.59, 17.48]), followed by the *rarely* (B=10.49, P<0.001; 95% CI [5.72, 15.27]), *sometimes* (B=9.42, P<0.001; 95% CI [4.71, 14.13]) and *often* groups (B=7.08, P=0.011; 95% CI [1.62, 12.53]). The non-overlapping CIs indicated that the effect size decreased as the frequency of interaction increased.

## Discussion

This study found that the PCPs in Selangor, Malaysia, held a generally negative perception towards patients with SUDs, with a mean DDPPQ score of 45.38 (interpolated to 70.76 on the 140-point scale). This finding is comparable to the mean score of 70.9 reported among acute care staff by Howard and Holmshaw^26^ and aligns with numerous reports from both Western^7,27-29^ and Eastern^8-10,30^ countries that have documented negative attitudes among healthcare workers.

**Table 3:** Predictors of the PCPs’ perception in the general linear model (N=233).

Variable		B	P-value	95% confidence interval	
				Lower bound	Upper bound
Experience with MMT	Yes	-4.91	**<0.001** ^**^	-6.9	-2.91
	No	0.00^a^		46 (7)	
	Never	12.53	**<0.001** ^**^	7.59	17.48
Frequency of seeing patients with SUDs	Rarely	10.49	**<0.001** ^**^	5.72	15.27
	Sometimes	9.42	**<0.001** ^**^	4.71	14.13
	Often	7.08	**0.011** ^*^	1.62	12.53
	Always	0.00^a^			

a Reference group

* P<0.05

** P<0.005

R^2^ = 0.268 Model assumptions were met; there were no interactions between the independent variables and no multicollinearity problems. MMT: Methadone maintenance therapy PCP: Primary care practitioner SUD: Substance Use Disorder

The findings contrast with other reports that show more favourable perceptions, particularly among emergency department staff and nurses in hospital settings.^14,17,31-37^ Such variations may be attributable to differences in clinical roles, training levels and the frequency of exposure to patients with SUDs. In Malaysia, societal stigma against individuals with SUDs, often driven by cultural and religious beliefs that frame addiction as a moral failure, remains prevalent.^8,38^ This stigma may be internalised by healthcare providers and amplified in primary care settings where exposure to SUD cases is less frequent.

Our analysis identified two significant factors associated with more positive perceptions: a higher frequency of interaction with patients with SUDs and professional experience with MMT. The finding that increased contact was linked to more positive attitudes is consistent with a report on Australian nurses.^36^ Extended contact allows PCPs to gain a deeper understanding of the complexities of addiction, fostering greater empathy. Conversely, a US study found that doctors spending over 40 hours per week on patient care had worse perceptions,^39^ although this discrepancy may be due to differences in the patient population and the historical context of the HIV epidemic.

Experience with MMT was also a strong predictor of more favourable perceptions. PCPs experienced in MMT likely have more frequent contact with patients with SUDs and a deeper understanding of harm-reduction principles. This aligns with the findings of a Canadian study, where reluctance to participate in MMT was linked to a lower knowledge level and an abstinence-only orientation.^40^ Limited knowledge and training contribute to healthcare workers feeling ill-equipped to manage SUDs effectively.^7^ Numerous studies have confirmed that education and training can significantly improve healthcare professionals’ attitudes and perceived knowledge,^7,26^ underscoring the need for comprehensive addiction training in primary care.

### Strengths

This study addresses a critical research gap in Malaysia by providing foundational insights into the perceptions of PCPs towards patients with SUDs. The findings serve as a valuable baseline for future comparative investigations and can inform the development of targeted educational interventions. Furthermore, the validated DDPPQ-M is a robust tool that can be utilised in future research to deepen the understanding of healthcare professionals’ perspectives in this field.

### Limitations

This study has several limitations. First, its cross-sectional design precludes the establishment of causal relationships. Second, the use of convenience sampling from selected clinics in Selangor limits the generalisability of the findings to all PCPs in the region. Third, selection bias may be present, as participants with limited experience in managing SUDs might have different perceptions. Finally, the DDPPQ treats drug users as a homogeneous group, which is a recognised limitation of the instrument. In future studies, researchers could develop and validate modified versions of the scale for specific substances.

### Recommendations

Based on the findings, we recommend the implementation of structured, continuous training and targeted awareness programmes to address the challenges of SUD management in primary care. These initiatives should aim to enhance providers’ knowledge, clinical competencies and confidence. We also recommend the strategic inclusion of all relevant healthcare personnel in MMT clinics through a rotational system to deepen their understanding of harm-reduction principles and reduce stigma. Previous research has shown that multicomponent interventions, including motivational interviewing, communication skills training and structured contact with individuals in recovery, are effective in improving provider attitude.^7,20^

## Conclusion

The PCPs in this study demonstrated a predominantly negative perception towards patients with SUDs. This negative perception was more pronounced among the PCPs with limited or no experience with MMT and those who had less frequent interaction with patients with SUDs. These findings highlight a critical need for enhanced training in addiction medicine, increased opportunities for clinical exposure to patients with SUDs and the promotion of a multidisciplinary, harm-reduction approach to care. Addressing societal stigma through broader public health initiatives would further support these efforts.

## Data sharing statement

The datasets utilised and/or analysed in this study can be obtained from the corresponding author upon reasonable request.

**How does this paper make a difference in general practice?**

- This study offers important insights into the perceptions of primary care practitioners (PCPs) towards patients with substance use disorders (SUDs) and their associated factors, which have been underexplored locally.
- SUDs are a pressing public health issue, and PCPs often serve as the first contact points for affected individuals. Understanding their perceptions is essential for designing effective interventions and improving patient outcomes.
- The study provides a useful baseline for future research and comparisons.
- The translated and validated tool (Drug and Drug Problems Perception Questionnaire- Malay version) is a valuable resource for studying healthcare professionals’ attitudes towards patients with SUDs in future research.
